# Early vigabatrin augmenting GABA-ergic pathways in post-anoxic status epilepticus (VIGAB-STAT) phase IIa clinical trial study protocol

**DOI:** 10.1186/s42466-022-00168-x

**Published:** 2022-01-24

**Authors:** Carolina B. Maciel, Fernanda J. P. Teixeira, Katie J. Dickinson, Jessica C. Spana, Lisa H. Merck, Alejandro A. Rabinstein, Robert Sergott, Guogen Shan, Guanhong Miao, Charles A. Peloquin, Katharina M. Busl, Lawrence J. Hirsch

**Affiliations:** 1 Division of Neurocritical Care, Department of Neurology, University of Florida College of Medicine, Gainesville, FL 32611 USA; 2 Department of Neurology, Yale University School of Medicine, New Haven, CT 06520 USA; 3 Department of Neurology, University of Utah, Salt Lake City, UT 84132 USA; 4 Department of Emergency Medicine, University of Florida College of Medicine, Gainesville, FL 32603 USA; 5 Department of Neurology, Mayo Clinic College of Medicine, Rochester, MN 55905 USA; 6 Department of Ophthalmology, Thomas Jefferson University, Philadelphia, PA 19107 USA; 7 Department of Biostatistics, University of Florida, Gainesville, FL 32603 USA; 8 Department of Pharmacotherapy and Translational Research, University of Florida College of Pharmacy, Gainesville, FL 32603 USA; 9 Department of Neurology, University of Florida, L3-100, 1149 Newell Drive, Gainesville, Florida 32610 USA

**Keywords:** Cardiac arrest, Heart arrest, Post-anoxic status epilepticus, GABA, Vigabatrin, Status epilepticus, Biomarkers

## Abstract

**Background:**

Nearly one in three unconscious cardiac arrest survivors experience post-anoxic status epilepticus (PASE). Historically, PASE has been deemed untreatable resulting in its exclusion from status epilepticus clinical trials. However, emerging reports of survivors achieving functional independence following early and aggressive treatment of PASE challenged this widespread therapeutic nihilism. In the absence of proven therapies specific to PASE, standard of care treatment leans on general management strategies for status epilepticus. Vigabatrin—an approved therapy for refractory focal-onset seizures in adults—inhibits the enzyme responsible for GABA catabolism, increases brain GABA levels and may act synergistically with anesthetic agents to abort seizures. Our central hypothesis is that early inhibition of GABA breakdown is possible in the post-cardiac arrest period and may be an effective adjunctive treatment in PASE.

**Methods:**

This is a phase IIa, single-center, open-label, pilot clinical trial with blinded outcome assessment, of a single dose of vigabatrin in 12 consecutive PASE subjects. Subjects will receive a single loading dose of 4500 mg of vigabatrin (or dose adjusted in moderate and severe renal impairment) via enteric tube within 48 h of PASE onset. Vigabatrin levels will be monitored at 0- (baseline), 0.5-, 1-, 2-, 3-, 6-, 12-, 24-, 48-, 72- and 168-h (7 days) post-vigabatrin. Serum biomarkers of neuronal injury will be measured at 0-, 24-, 48-, 72- and 96-h post-vigabatrin. The primary feasibility endpoint is the proportion of enrolled subjects among identified eligible subjects receiving vigabatrin within 48 h of PASE onset. The primary pharmacokinetic endpoint is the measured vigabatrin level at 3 h post-administration. Descriptive statistics with rates and proportions will be obtained regarding feasibility outcomes, along with the noncompartmental method for pharmacokinetic analyses. The area under the vigabatrin concentration-time curve in plasma from zero to the time of the last quantifiable concentration (AUC_0-tlqc_) will be calculated to estimate dose-linear pharmacokinetics.

**Perspective:**

Vigabatrin demonstrates high potential for synergism with current standard of care therapies. Demonstration of the feasibility of vigabatrin administration and preliminary safety in PASE will pave the way for future efficacy and safety trials of this pharmacotherapeutic.

*Trial Registration* NCT04772547.

**Supplementary Information:**

The online version contains supplementary material available at 10.1186/s42466-022-00168-x.

## Introduction

Cardiac arrest claims millions of lives annually across the globe. Outcomes depend on resuscitation efforts and on the severity of global brain injury, which is comprised of primary insult (accrued during circulation standstill) and ongoing injury due to gaps between the energetic supply and demand from the brain (secondary brain injury). Once return of spontaneous circulation (ROSC) occurs, treatment relies on mitigating cerebral energy demand. Seizures—synchronized electrical phenomena produced by cortical neurons—are common after cardiac arrest and lead to a marked increase in cerebral metabolic activity, and hence incur a great risk for secondary injury. In fact, one in three unconscious cardiac arrest survivors that undergo targeted temperature management experience post-anoxic status epilepticus (PASE). Recent evidence suggests that early and aggressive treatment of PASE may not only lead to improved survival, but also to an increased likelihood of functional independence [1].

Historically, PASE has been linked to nearly universal mortality [2]; however, the cardiac arrest literature is confounded by bias arising from a decreased likelihood in the provision of aggressive supportive care when poor outcomes are expected. As a result, this population has been traditionally excluded from observational studies and clinical trials of status epilepticus, and the management of these patients varies widely. Despite increasing awareness of the potential positive impact of aggressive treatment of PASE with reports of neurologically intact survival [1–3], structured therapeutic interventions remain largely unexplored in this population. Consequently, conventional treatment algorithms are extrapolated from other settings [4], such as convulsive status epilepticus guidelines [5, 6]. These approaches include parenteral benzodiazepines as first-line therapy (e.g., lorazepam, midazolam), followed by a loading dose of a parenteral conventional antiseizure medication (e.g., fosphenytoin, valproic acid, levetiracetam, lacosamide) as second-line therapy, and induced coma with general anesthetic infusion as third-line therapy (e.g., midazolam, propofol, pentobarbital, ketamine). Recent pre-clinical studies have demonstrated the potential benefits of a combined therapeutic strategy in status epilepticus aiming for synergistic mechanisms of action, which may reduce exposure to anesthetics and mitigate their associated morbidity.

**Fig. 1:**
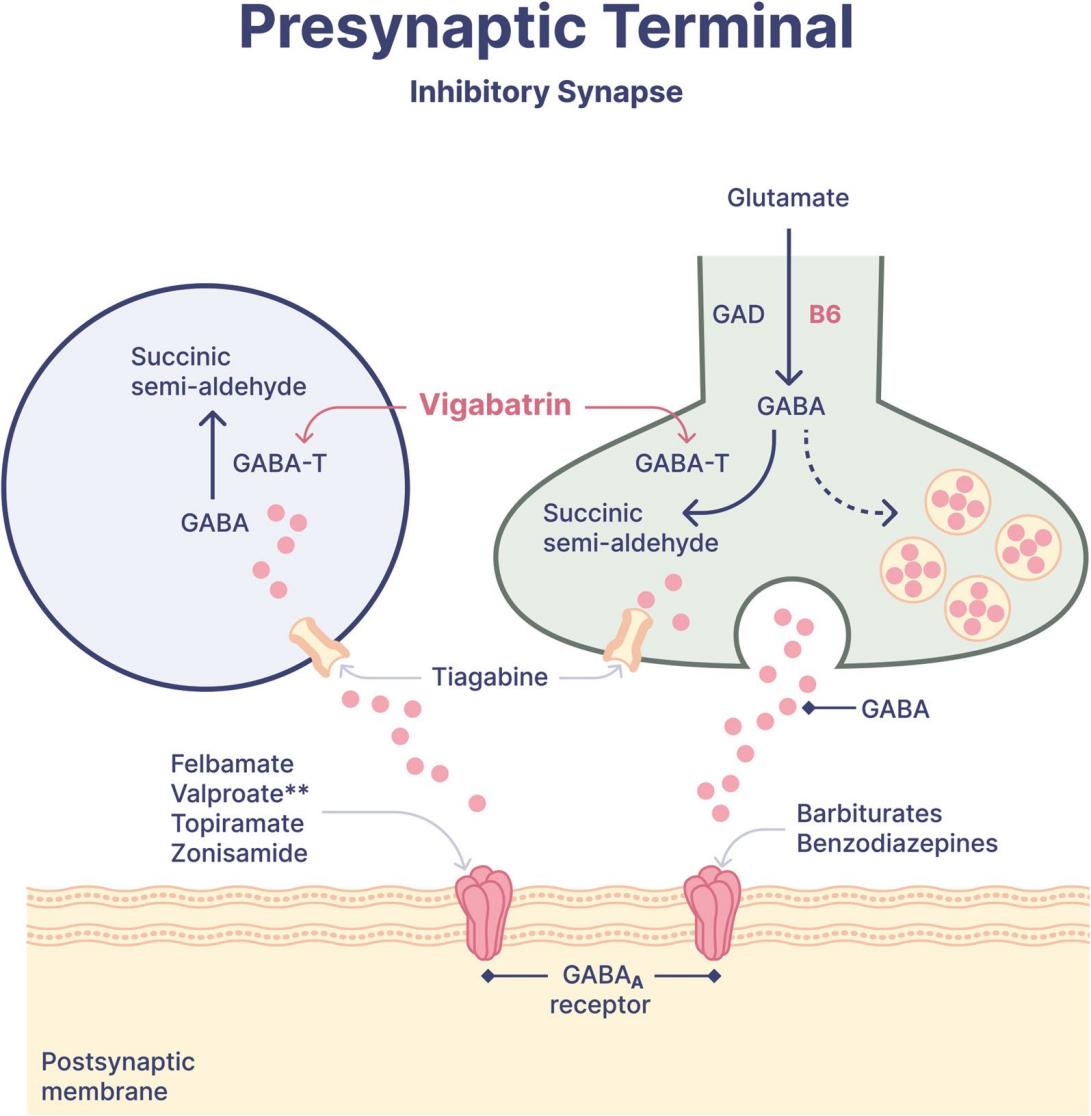
Vigabatrin mechanism in the inhibitory synapse

During the course of status epilepticus, there is marked alteration of gamma-aminobutyric acid (GABA) metabolism; the rate of GABA synthesis decreases GABA turnover time increases up to 3-fold [7], and cell surface GABA receptors migrate to the intracellular space [8]. Conventional first-line therapy acts by enhancing the GABAA receptor to increase its inhibitory tone. Vigabatrin, 4-amino-5-hexenoic acid or gamma-vinyl GABA, is a structural analog of GABA and irreversibly inhibits GABA-transaminase—the enzyme responsible for GABA catabolism, thereby increasing brain levels of GABA (Fig. 1) [9]. Vigabatrin may also stimulate GABA release [10]. The net effect is a significant elevation in GABA levels in nerve terminals, which facilitates GABA-mediated synaptic transmissions and leads to a marked and sustained antiseizure effect. The efficacy of vigabatrin as an adjunctive therapy for intractable focal epilepsy is well-established in the literature [11]. Vigabatrin may also potentiate the therapeutic effects of GABA-ergic anesthetics and other antiseizure medications [12] that are commonly used in the post-cardiac arrest period for either sedation, seizure control or symptomatic control of myoclonus (e.g., propofol, midazolam, and valproate). In addition to its antiseizure effect, vigabatrin may also mitigate ischemic-reperfusion injury by restoring the balance between GABA and glutamate transmissions [13].

Currently, vigabatrin is an FDA-approved therapy for infantile spasms and refractory focal-onset seizures in adults. It is also being investigated as a neuroprotective therapy in traumatic brain injury [14]. Its role as an adjunctive therapy in status epilepticus has a strong scientific basis but has not been clinically established thus far. Furthermore, while the pharmacokinetics of vigabatrin absorption have been demonstrated in healthy volunteers and in patients with refractory epilepsy in the outpatient setting, pharmacokinetic data in the critically ill are scarce.

### Aim

The aim of this study is to demonstrate the feasibility of administering a single enteral loading dose of vigabatrin within 48 h of PASE onset in unconscious cardiac arrest survivors and characterize its absorption, regardless of location of cardiac arrest (i.e., out-of-hospital versus in-hospital) and type of non-perfusing rhythm (i.e., asystole, pulseless electrical activity, pulseless ventricular tachycardia or ventricular fibrillation).

### Hypothesis

Our central hypothesis is that augmenting GABA-ergic pathways by early inhibition of GABA breakdown may be an effective adjunctive treatment for PASE by working synergistically with GABA-ergic anesthetics commonly used in post-cardiac arrest period.

## Methods

### Study design

A phase IIa, single-center, open-label pilot clinical trial with blinded outcome assessment, of early administration of a single 4500 mg (or dose adjusted in moderate and severe renal impairment) dose of vigabatrin in 12 consecutive PASE subjects. This study protocol is prepared according to the SPIRIT 2013 statement and the SPIRIT-PRO Extension (see Additional file 1: Table S1).

### Screening

Participants will be recruited at the University of Florida Health Shands Hospital Emergency Department and intensive care units (ICUs). The principal investigator and co-investigators will screen all successfully resuscitated cardiac arrest individuals undergoing continuous electroencephalogram (cEEG) for PASE development at least four times daily at regular intervals (i.e., every 4-6 h). PASE is defined according to American Clinical Neurophysiology Society criteria for electrographic status epilepticus: any pattern displaying definite evolution or epileptiform discharges averaging > 2.5Hz lasting ≥ 10 continuous minutes or comprising ≥ 20% of any 60-minute period of recording [15]. The window from PASE onset to drug delivery of 48 h is limited, particularly when accounting for the time to obtain consent and enrollment in the Risk Evaluation and Mitigation Strategy (REMS) program from a legally authorized representative is pursued in the acute setting and enteral access for drug delivery is required. To expedite the process, we will approach the legally authorized representatives of potential participants for consent as soon as interictal epileptiform activity is identified on EEG as these subjects are at high risk for subsequent PASE [16]. We will maintain a screening log to keep track of all eligible subjects, which will inform future decisions on inclusion/exclusion criteria in the subsequent study phases.

### Eligibility criteria

PASE may occur following any type of cardiac arrest, regardless of the location of its occurrence (i.e., out-of-hospital versus in-hospital), the type of non-perfusing rhythm (i.e., asystole, pulseless electrical activity, pulseless ventricular tachycardia or ventricular fibrillation) or treatment with targeted temperature management. Individuals meeting the eligibility criteria displayed in Table 1 will be approached. Those with non-traumatic cardiac arrest will be eligible if the decision to initiate and maintain care aimed at preservation of life in the early post-cardiac arrest period and treat unequivocal electrographic status epilepticus has been made.

**Table 1:** Eligibility criteria

Eligibility criteria	
*Inclusion*	
1. 18–80 years of age	The target study population is adults, hence ≥ 18 years. Individuals > 80 years have a lower likelihood of successfully tolerating aggressive treatment for PASE, including escalating doses of intravenous anesthetic infusions, and are subjected to high rates of withdrawal of life sustaining therapy in the early post-cardiac arrest period. The upper age limit of 80 years has been used in other cardiac arrest trials
2. Non-traumatic cardiac arrest of any rhythm, etiology and location of occurrence	PASE may occur following cardiac arrest of any type, regardless of the location of its occurrence (out-of hospital vs in-hospital) or the type of non-perfusing rhythm. Traumatic cardiac arrests have different organ injury and clinical trajectories than non-traumatic cardiac arrests
3. Requiring anesthetic infusion for any indication	To standardize starting point of anesthetic infusions prior to initiation of therapeutic algorithm for PASE
4. Arterial access for frequent blood sampling	Facilitate timely and standardized collection of blood sampling for pharmacokinetic analyses
5. Established enteral access within 48 h of PASE onset	There is currently no parenteral form of vigabatrin, thus, enteral access is a prerequisite for participation
*Exclusion*	
1. Prior history of generalized epilepsy	Vigabatrin may exacerbate certain types of primary epilepsy such as primary generalized epilepsy
2. Gastrointestinal surgery within the last 21 days	Avoid confounding effect that could affect absorption of study drug
3. Pregnancy	There is currently no controlled data verifying the safety of vigabatrin dosing in pregnant women, so they are excluded from this study
4. PASE onset preceding initiation of EEG monitoring	If PASE onset occurred prior to the initiation of monitoring, the start time for the onset of PASE cannot be determined

### Blinding

This will be an open label study. EEG recordings and data pertaining to screening for vigabatrin-related visual loss will be centrally analyzed by investigators blinded to the timing of vigabatrin administration, details of arrest and drug levels (LHJ and RS, respectively). De-identified EEG recordings will be exported to Yale University for central analyses, and de-identified Visual Field Perimetry studies will be exported to Tomas Jefferson University. Clinical outcomes will be obtained by trained study personnel using validated scales and blinded to the timing of vigabatrin administration, details of cardiac arrest, and drug levels.

### Standardized care

**Fig. 2:**
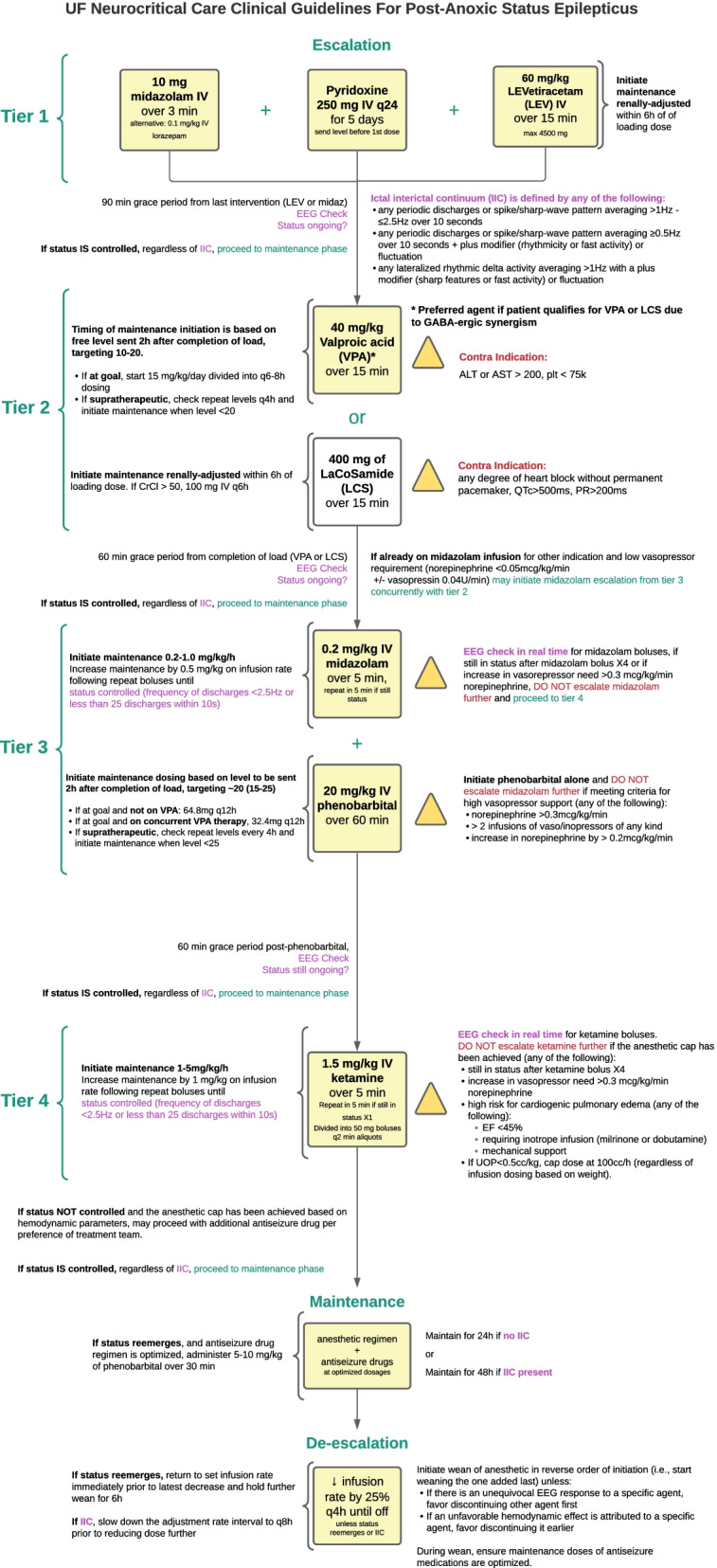
University of Florida neurocritical care guidelines for post-anoxic status epilepticus

General critical care will be standardized to the extent possible for all subjects. Management of fluid homeostasis, hemodynamics, respiration, metabolic disturbances, and seizures will be according to local protocols and standard of care. Long-term monitoring with continuous EEG will be initiated as soon as possible following ROSC and maintained for at least 72 h for all unconscious cardiac arrest survivors or 24 h post-discontinuation of anesthetics in those who develop post-anoxic status epilepticus per institutional practice. The therapeutic approach to PASE has been standardized at the University of Florida and independent of this study protocol as standard of care based on available guidelines [4–6] and tailored to the post-cardiac arrest period: risk mitigation of detrimental hemodynamic effects and prioritization of synergistic GABA-ergic pathway are the mainstay of the treatment protocol (Fig. 2). Diversions from the algorithm may occur at the discretion of the treating neurointensivist and will be documented in study records.

### Intervention

Subjects will receive a one-time loading dose of 4500 mg of vigabatrin in powder for oral solution formulation via enteric tube within 48 h of PASE onset. Subjects with moderate renal impairment (CrCl 30–50 mL/min) will receive 2250 mg of vigabatrin (50% dose reduction), and subjects with severe renal impairment (CrCl < 30 mL/min) will receive 1125 mg of vigabatrin (75% dose reduction). Furthermore, the total volume administered will be dictated by the dose of vigabatrin (10 mL of water for each 500mg packet). A single vigabatrin load is sufficient to achieve a prolonged GABA catabolism inhibition and minimizes the risk of vision loss associated with chronic exposure. The inhibition of central and peripheral GABA-t has been shown to outlast serum vigabatrin elimination [9]. A dose ceiling effect has been found in initial studies of vigabatrin, in which doses > 60 mg/kg failed to elevate cerebral GABA levels further [17], and prolonged elevations of cerebral GABA levels may reduce GABA synthesis [17]. Thus, to minimize toxicity risk, maximize synergistic potential with co-administration of GABA-ergic anesthetics and the therapeutic window, we selected a single maximum loading dose of 4500 mg for those with preserved renal function. If subjects are receiving the study drug through a naso- or orogastric tube, or gastrostomy tube that is ordered to be connected to suction, the tube will remain clamped for a minimum of 30 minutes. Vigabatrin will not be co-administered with other enteral drugs, and no drugs will be given  ± 15 min of vigabatrin administration when possible, per standard of care. The dose will be administered in parallel with standard antiseizure therapy, regardless of resolution or control of electrographic status epilepticus at time of vigabatrin administration.

### Pharmacokinetic sampling

Total plasma concentrations of vigabatrin will be measured in the Infectious Diseases Pharmacokinetics Lab (IDPL) at the University of Florida, using a validated liquid chromatography with tandem mass spectrometry assay. The range of detection is 0.50–100.00 mg/L and the intra- and inter-batch precision and accuracy were < 4% during validation tests. Vigabatrin concentrations will be monitored serially at baseline (prior to load), 0.5 h (± 5 min), 1 h (± 5 min), 2 h (± 15 min), 3 h (± 15 min), 6 h (± 15 min), 12 h (± 15 min), 24 h (± 30 min), 48 h (± 30 min), 72 h (± 30 min), and 7 days or 168 h (± 30 min) post-vigabatrin administration along with daily levels of concurrent antiseizure medications. All pharmacokinetic samples will be collected from an arterial line. If sufficient arterial access is not available at the scheduled time, venous sampling from a central venous catheter will be the preferred second method of blood collection and will be noted in source documentation. Our study follows a design used in other studies, measuring serial vigabatrin concentrations at baseline, 1 h, 3 h, and 12 h following vigabatrin administration [14], the addition of 0-, 0.5-, 1-, 2-, 3-, 6-, 12-, 24-, 48-, 72- and 168-h (7 days) level measurements is aimed at accurately describing absorption, confirming clearance of the drug, and confirming the reported lack of correlation of drug levels with a sustained clinical effect. The pre-dose sample allows for confirmation that vigabatrin is not already present in the study subjects and no interference in the quantification method.

### Neuronal injury biomarkers

Biomarkers of neuronal injury including neuron-specific enolase (NSE), glial fibrillary acidic protein (GFAP), ubiquitin C-terminal hydrolase L1 (UCH-L1), neurofilament light chain protein (NfL) and tau protein will also be measured in the serum at five timepoints including at baseline, 24 h, 48 h, 72 h and 96 h post-vigabatrin administration.

### Clinical variables

We will collect baseline data on subjects, including National Identification number, demographics, medical history, home medications, factors predisposing to seizures (e.g., prior traumatic brain injury or other structural injury, history of epilepsy, congenital malformations), standard of care laboratory data (including comprehensive metabolic panel and blood counts), and pre-hospital drugs administered. We will collect neurological assessment and Full Outline of Unresponsiveness score (FOUR) prior to EEG monitoring, during ICU phase, at ICU discharge, and at hospital discharge. Clinical signs suggestive of seizures will be collected prior to EEG monitoring during hospital admission. Throughout hospitalization, we will collect adverse events (AEs), code status and changes in the level of medical interventions, and antiseizure medication dosing. Neurologic assessments including pupillometry measurements will be collected 1-h post-vigabatrin and daily for 7 days thereafter. At ICU discharge, we will collect duration of mechanical ventilation and length of ICU stay. At hospital discharge, we will also collect discharge status, discharge destination, and Epidemiology-based Mortality Score in Status Epilepticus. Long-term follow up will be obtained at 180 days after intervention; we will collect survival status, modified Rankin scale (mRS), Glasgow–Pittsburgh Cerebral Performance Category Extended scale (CPC-E), Glasgow Outcome Scale Extended (GOS-E), Montreal Cognitive Assessment (MoCA), and The Short Form 36 Health Survey Questionnaire (SF-36). Given the low rates of survival to hospital discharge associated with PASE [1], we expect that long-term follow-up data will not be available in 80% of the cohort.

### Visual loss surveillance

Vigabatrin has been associated with concentric visual defect from retinopathy; however, this has only been reported with prolonged vigabatrin exposure [18]. Taurine depletion from vigabatrin has been implicated as a potential mechanism as taurine deficiency has been associated with a higher retinopathy risk [19]. We will monitor taurine levels at baseline, 72 h, and 7 days post-vigabatrin. In subjects who regain consciousness and survive to hospital discharge, clinical assessments of visual loss will include Goldmann visual field perimetry and Visual Function Questionnaire 25. Given the high mortality and high prevalence of severe disorder of consciousness associated with PASE [1], both of which precluding the administration of Goldmann visual field perimetry and the Visual Function Questionnaire 25, we expect that these data will be available in < 20% of the cohort.

### Electrophysiologic variables

To explore the biologic effects of early GABA-ergic augmentation with vigabatrin, central analysis of EEG data will include adjudication of time of PASE onset, time of PASE control (defined as the end of the first hour without unequivocal status epilepticus, regardless of concurrent anesthetic therapy), time of PASE resolution (defined as the end of the first hour after successful wean of anesthetic infusion to non-status epilepticus doses without unequivocal status epilepticus). As intravenous anesthetics may be required for indications other than status epilepticus in intubated patients, the following thresholds are considered status epilepticus doses of anesthetics: (1) midazolam ≥ 0.1 mg/kg/h, (2) propofol ≥ 50 mcg/kg/min, and (3) ketamine ≥ 1 mg/kg/h. Additional EEG variables include characterization of features of background and rhythmic and periodic patterns.

### Data management

Individual subject data will be handled according to Health Insurance Portability and Accountability Act and institutional policy. All subject data will be collected in a REDCap database and will be de-identified, with assigned unique identifiers. Raw EEG recordings and Visual Field Perimetry will be de-identified.

### Primary and secondary outcomes

The primary outcomes of interest are feasibility of vigabatrin administration within 48 h of PASE onset and subsequent absorption of the drug. We will track the ratio between identified eligible and enrolled subjects and assess reasons for decline in study participation. We will track the proportion of subjects in whom PASE was present upon connection to EEG and the proportion of enrolled subjects who received a vigabatrin load within 12 and 24 h of PASE onset. We will explore the potential feasibility of ultra-early administration of vigabatrin in future study phases. In addition, by analyzing serial vigabatrin levels, we will characterize drug elimination. Secondary outcomes include time to PASE detection, ultra-early (within 12 h of PASE onset) drug delivery and rates of visual screening completion in survivors who regained consciousness. Exploratory outcomes include frequency of abnormal visual fields and taurine deficiency following vigabatrin, time to PASE control and resolution, and weight-based cumulative anesthetic infusion for PASE.

### Statistical analysis

This feasibility pilot is not intended to detect therapeutic effects. Thus, we have omitted power calculations. Descriptive statistics with rates and proportions will be obtained regarding feasibility outcomes, as well as demographic and clinical outcomes. Missing data will be reported. Regarding the pharmacokinetic outcome, we will use the noncompartmental method for pharmacokinetic analyses. The area under the vigabatrin concentration-time curve in plasma from zero to the time of the last quantifiable concentration (AUC_0-tlqc_) will be calculated using the linear trapezoidal methods, and the AUC will be extrapolated to infinity for the evaluation of AUC_0-inf_.

## Perspective

The explicit purpose of this feasibility study is to deliver vigabatrin within 48 h of PASE onset and have measurable levels by 3–12 h post-load in addition to completing visual screening safety assessments in subjects who survive the index hospitalization and regain consciousness. The reported bioavailability of vigabatrin is 100%, with a wide volume of distribution, minimal binding to blood proteins and elimination mainly through renal clearance. While plasma concentrations of vigabatrin do not correlate with its effectiveness [20], they confirm successful absorption of this drug. Landmark vigabatrin trials did not include critically ill subjects [11], thus the demonstration of absorption in this patient population is imperative prior to proceeding with large scale dose finding trials. Several factors may affect vigabatrin absorption in the post-cardiac arrest period, such as continuous enteral feeding, decreased mesenteric blood flow due to vasopressor drug use, and decreased bowel motility due to neuromuscular blockade. Further, impaired clearance may also occur due to targeted temperature management, renal impairment, and high prevalence of older individuals in this population.

A dose ceiling effect has been found in initial studies of vigabatrin, in which doses >60mg/kg failed to elevate cerebral GABA levels further, and prolonged elevations of cerebral GABA levels may reduce GABA synthesis [17]. In an individual ~ 70 kg, 60 mg/kg corresponds to 4200 mg. Therefore, the chosen single vigabatrin load is expected to be sufficient to achieve a prolonged GABA catabolism inhibition while minimizing the risk of vision loss associated with chronic exposure. Later study phases will assess the potential therapeutic effects of this intervention.

By identifying the potential for a novel indication of a drug with high potential for synergism in PASE, this study is the stepping-stone to filling an important knowledge gap in the care of cardiac arrest survivors.

## Supplementary Information

**Additional file 1: Table S1.** Schedule of enrolment, interventions, and assessments. Time points: - T2: at onset of PASE; - T1: within 48h of PASE onset and before administration of VGB; T0: administration of VGB within 48h of PASE onset; T13: ICU discharge; T14: hospital discharge; T15:180 days after PASE onset. Abbreviations: VGB: Vigabatrin; MRI: Magnetic Resonance Imaging; EMSE: Epidemiology-based Mortality Score in Status Epilepticus; mRS: modified Rankin scale; FOUR: Full Outline of Unresponsiveness score; MoCA: Montreal Cognitive Assessment; CPC-E: Glasgow–Pittsburgh Cerebral Performance Category Extended scale; GOS-E: Glasgow Outcome Scale Extended; SF-36: The Short Form 36 Health Survey Questionnaire; VQF-25: Visual Function Questionnaire 25.

## Data Availability

This study conforms to the open data policy of American Heart Association to deposit de-identified data into an approved repository within 12 months of study completion for independent verification of study results.

## Abbreviations

AEs: Adverse events
AUC: Area under the ROC curve
cEEG: Continuous electroencephalogram
CPC: Glasgow–Pittsburgh cerebral performance category scale
CrCl: Creatinine cleareance
EEG: Electroencephalogram
FOUR: Full outline of unresponsiveness
GABA: Gamma-aminobutyric acid
GABA-t: Gamma-aminobutyric acid transaminase
GFAP: Glial fibrillary acidic protein
ICU: Intensive care unit
MoCA: Montreal cognitive assessment
mRS: Modified Rankin scale
NfL: Neurofilament light chain protein
NSE: Neuron-specific enolase
PASE: Post-anoxic status epilepticus
REMS: Risk evaluation and mitigation strategies
ROSC: Return of spontaneous circulation
SF-36: Short form 36 health survey questionnaire
SPIRIT: Standard protocol items: recommendations for interventional trials
UCH-L1: Ubiquitin C-terminal hydrolase L1
