# Supplementary material for: Early vigabatrin augmenting GABA-ergic pathways in post-anoxic status epilepticus (VIGAB-STAT) phase IIa clinical trial study protocol
Source: Neurol Res Pract. 2022 Jan 24;4:4. doi: 10.1186/s42466-022-00168-x (PMC8785535; doi:10.1186/s42466-022-00168-x)
Supplement: Supplementary file 1 — Additional file 1: Table S1. Schedule of enrolment, interventions, and assessments. Time points: - T2: at onset of PASE; - T1: within 48h of PASE onset and before administration of VGB; T0: administration of VGB within 48h of PASE onset; T13: ICU discharge; T14: hospital discharge; T15:180 days after PASE onset. Abbreviations: VGB: Vigabatrin; MRI: Magnetic Resonance Imaging; EMSE: Epidemiology-based Mortality Score in Status Epilepticus; mRS: modified Rankin scale; FOUR: Full Outline of Unresponsiveness score; MoCA: Montreal Cognitive Assessment; CPC-E: Glasgow–Pittsburgh Cerebral Performance Category Extended scale; GOS-E: Glasgow Outcome Scale Extended; SF-36: The Short Form 36 Health Survey Questionnaire; VQF-25: Visual Function Questionnaire 25. [file 42466_2022_168_MOESM1_ESM.docx]

| Study Period | | | | | | | | | | | | | | | | | | |
| --- | --- | --- | --- | --- | --- | --- | --- | --- | --- | --- | --- | --- | --- | --- | --- | --- | --- | --- |
| Time Point | Enrollment | Pre-Allocation | Allocation | Post-Allocation (hours *after* VGB administration) | | | | | | | | | | | | | | |
|  | -T2 | -1T | T0 | T1  (0.5h) | T2  (1h) | T3 (2h) | T4  (3h) | T5  (6h) | T6  (12h) | T7  (24h) | T8 (48h) | T9 (72h) | T10 (96h) | T11  (120h) | T12 (168h) | T13 | T14 | T15 |
| **Enrollment:** |  |  |  |  |  |  |  |  |  |  |  |  |  |  |  |  |  |  |
| Eligibility Screen | X |  |  |  |  |  |  |  |  |  |  |  |  |  |  |  |  |  |
| Informed Consent | X |  |  |  |  |  |  |  |  |  |  |  |  |  |  |  |  |  |
| Allocation |  |  | X |  |  |  |  |  |  |  |  |  |  |  |  |  |  |  |
| **Intervention:** |  |  |  |  |  |  |  |  |  |  |  |  |  |  |  |  |  |  |
| VGB Administration |  |  | X |  |  |  |  |  |  |  |  |  |  |  |  |  |  |  |
| **Assessments:** |  |  |  |  |  |  |  |  |  |  |  |  |  |  |  |  |  |  |
| VGB Levels |  | X |  | X | X | X | X | X | X | X | X | X | X |  | X |  |  |  |
| Biomarkers |  |  | X |  |  |  |  |  |  | X | X | X | X |  |  |  |  |  |
| Taurine Levels |  | X |  |  |  |  |  |  |  |  |  | X |  |  | X |  |  |  |
| Anti-Seizure drug levels |  | X |  |  | X |  | X |  | X | X |  | X |  |  | X |  |  |  |
| Pupillometry |  | X | X |  | X |  |  |  |  | X | X | X | X | X | X |  |  |  |
| MRI Window |  |  |  |  |  |  |  |  |  |  |  | X | X | X |  |  |  |  |
| Neurological Assessment | X | X | X |  |  |  |  |  |  | X | X | X | X | X | X |  |  | X |
| EMSE |  |  |  |  |  |  |  |  |  |  |  |  |  |  |  |  | X |  |
| FOUR |  | X | X |  | X |  | X |  | X | X | X | X | X | X | X | X | X |  |
| MoCA |  |  |  |  |  |  |  |  |  |  |  |  |  |  |  |  |  | X |
| mRS, CPC-E, GOS-E, and SF-36 |  |  |  |  |  |  |  |  |  |  |  |  |  |  |  |  | X | X |
| VFQ-25 |  |  |  |  |  |  |  |  |  |  |  |  |  |  |  |  |  | X |
| Goldman Perimetry |  |  |  |  |  |  |  |  |  |  |  |  |  |  |  |  |  | X |

**Additional file 1**
